# Investigating solitude as a tool for downregulation of daily arousal using ecological momentary assessments

**DOI:** 10.1111/jopy.12939

**Published:** 2024-05-17

**Authors:** Thuy‐vy T. Nguyen, Delali Konu, Samuel Forbes

**Affiliations:** ^1^ Department of Psychology Durham University Durham UK

**Keywords:** emotion regulation, extraversion, neuroticism, solitude

## Abstract

**Objective:**

This research explored arousal levels as a motivating factor for solitude‐seeking. We hypothesized that solitude becomes more desirable when high‐arousal emotions were heightened and individual differences in extraversion and neuroticism would moderate this pattern.

**Method:**

We tracked individuals' hourly experiences throughout a day. We assessed their high‐arousal positive (e.g., excitement) and negative emotions (e.g., tension), whether they were alone or with others, and their preferred situation at the time of the signal. We gathered 4338 surveys from 362 participants, with 103 participants completing all hourly surveys.

**Results:**

Preference for and incidence of solitude changed throughout the day. Contrary to our hypotheses, lagged analyses did not indicate high‐arousal emotions predicting reports of being alone an hour later. However, individuals were more likely to express a preference for solitude while experiencing high‐arousal negative emotions, and less so while experiencing positive emotions. Younger individuals display stronger preference for solitude during experiences of high‐arousal negative emotions. Extraversion and neuroticism did not moderate these patterns.

**Conclusions:**

The results highlight the distinctive appeal of solitude as a space for young adults to deal with negative emotions. We discussed how these findings are connected to existing literature and implications for future research.

## 1 INTRODUCTION

In our daily life, we find ourselves alone quite often. Solitude occurs spontaneously in between social times or sometimes might be pursued purposefully when we need some time for ourselves. Recent experimental studies have showed that solitude might function as an “approach for affective self‐regulation” (Nguyen et al., 2018), particularly allowing opportunities to downregulate high‐arousal emotions like excitement or anger. Building from that framework, the present research aims to capture occurrences of solitude in daily life to test whether solitude is more likely to occur when emotional activation is high.

### 1.1 Conceptualizations of solitude

In previous research, solitude is commonly defined as either a physical (Larson, 1990; Larson & Csikszentmihalyi, 2014) or subjective state of being alone (Lay et al., 2019, p. 20; Long et al., 2003; Pauly et al., 2018). This phenomenon can be observed when people are by themselves in a private space such as their own home, or when they sit alone without interacting with those around them in a public space such as a library or a park. Long and Averill (2003) offered a conceptualization that encompass all those situations of solitude: “a state characterised by disengagement from the immediate demands of other people.” This conceptualization excludes situations when someone might be alone but interact remotely with others on the phone, which is consistent with what Campbell and Ross (2021) recently suggested that communication through devices or online needs to be factored out of our conceptualization of solitude.

Under the umbrella of this conceptualization, several approaches have been used to operationalize solitude in quantitative research. In experimental studies, researchers observed solitude by having participants spend time alone physically (with no one around) (Nguyen et al., 2018, 2022; Rodriguez et al., 2020). In studies that used self‐reported surveys, researchers used specific criteria, such as whether the person was alone physically, whether they were interacting with someone, etc., to categorize data into either alone or not‐alone situations (Lay et al., 2019; Pauly et al., 2017; Thomas et al., 2021).

The above conceptualization separates solitude from loneliness; this distinction is accepted by both researchers and laypeople (Galanaki, 2004; Weinstein et al., 2022). It also means that solitude is studied as a neutral concept and it is different from “positive solitude” proposed by Ost Mor et al. (2020). Finally, people might also approach solitude with different types of motivation (Thomas & Azmitia, 2019); however, the reason why someone spends time alone is not considered to be part of the conceptualization of solitude (Nguyen et al., 2021).

For the present research, we used ecological momentary assessments to track situations when people were alone or with other people. We adopted the broad conceptualization of solitude that captures both physical and subjective aspects. To operationalize it for this research purpose, we endorsed Pauly et al.'s (2018) and Lay et al.'s (2019) measures of momentary solitude; that is, a time when a person does not interact with anyone, regardless of whether the person is physically alone or surrounded by other people. This operationalization aligns with both conceptualizations proposed by Long and Averill (2023) and Campbell and Ross (2021).

### 1.2 Arousal regulation mechanism of solitude

Across the lifespan, previous data suggested that on average, working adults spend about a third of their day alone (Larson, 1990). This is consistent with recent data we collected from the US and UK (via YouGov), which shows that most adults spend about 4 to 6 h a day in solitude (not interacting with other people either remotely, on the Internet, or in person). While adults' experiences with solitude might vary depending on how much and how often they spend time in this state, solitude seems to have one salient significance in people's lives: a study of 18,000 adults around the world showed that most adults nominated time spent alone as one of the top activities that they do for rest (Hammond, 2016).

Psychological research could reveal insights into the mechanisms underlying the restful nature of solitude. Our moods change in a unique way when we spend time alone. Evidence gathered from Ecological Momentary Assessment studies showed that positive emotions high on arousal levels such as being excited or energized were rated lower when people spend time alone (Pauly et al., 2017). Previous research interpreted the drop of those positive emotions to mean that solitude was associated with lower mood like being drowsy and lonely (Larson et al., 1980; Larson & Csikszentmihalyi, 2014). However, a different perspective on the effect of solitude on daily mood was introduced when Nguyen et al. (2018) captured emotional states using the “circumplex” model of affect with two dimensions: the pleasant–unpleasant dimension, and the activation dimension. Using this model, emotional states can be categorized into four types: high‐arousal positive affect, high‐arousal negative affect, low‐arousal positive affect, and low‐arousal negative affect.

In four original experiments (Nguyen et al., 2018) and two recent replications (Nguyen et al., 2022), the researchers showed that sitting alone for a brief 15 min consistently led to drops in high‐arousal types of affect, both positive emotions such as being excited or alert, as well as negative emotions like being angry and worried. Additionally, while participants in the lab felt less excited and alert, they gained a different type of positive affect, one that is lower in arousal like feeling relaxed and calm. These experimental findings were consistent with previous evidence from experience sampling studies showing the regulatory function of solitude. For example, in an adolescent sample, (Larson & Csikszentmihalyi, 1978) showed that mood was improved in the next social interactions after participants spent some time in solitude. Therefore, in combination with the evidence that many adults around the world see solitude as a time for rest, psychological research suggests that solitude allows rest through downregulation of high‐arousal emotions. As such, we proposed that this role of solitude in arousal regulation processes might be the key to maintaining balance between social time and solitary time (Coplan et al., 2019; Weinstein et al., 2022).

### 1.3 Situational driver of daily solitude

Generally, previous research shows that when solitude is chosen or motivated for self‐determined reasons (e.g., for creative pursuits, seeking peace, and engaging in self‐discovery) (Thomas & Azmitia, 2019), its benefits are heightened, including more positive affect (Lay et al., 2018; Nguyen et al., 2018), greater experiences of meaningfulness and productivity (Tse et al., 2021). However, we have not yet understood when such desire and motivation for solitude arise: are there times during our days when we are more likely to seek solitude? Ren and colleagues (2016, 2021) have studied one scenario in which preference for solitude was heightened after being ostracized. In other words, preference for solitude could be a symptom of social withdrawal and motivated by the desire to avoid other people as sources of distress. However, there are many other occasions when people would like to have some time alone. For example, people might need quiet time to process difficult feelings after hearing bad news, or they might want a moment to hit the “reset” button before the next high‐arousal activity after many back‐to‐back interactions.

For the present research, we aimed to look at arousal as the driver that motivates solitude‐seeking. Deriving from the framework that solitude promotes rest through downregulation of high‐arousal emotions, we expected that solitude is more likely to occur when those high‐arousal emotions are heightened. To achieve this research aim, we tracked participants' experiences throughout the day to catch solitude “in the wild.” Based on previous experiments demonstrating that spending time alone led to drops in high‐arousal types of affect and increases in low‐arousal types of affect (Nguyen et al., 2018), this research used Ecological Momentary Assessment (EMA) to examine whether experiences of emotional states that vary on both activation and pleasant/unpleasant dimension would more likely precede time spent alone than with others. *We predicted that people would be more likely to be alone* (*instead of interacting with others*) *after experiencing high‐arousal emotions assessed at a previous time point*. At Stage 1 of this registered report, we thought it was possible that this would be particularly true for high‐arousal negative emotions. If so, it would be consistent with the findings by Ren et al. (2016, 2021) that solitude is more preferred after being ostracized. Additionally, to account for situational constraints that might prevent someone to seek solitude even when they might prefer doing so, we also looked at increased preference for solitude as a function of heightened arousal levels throughout the day.

### 1.4 Person–situation interaction

Beyond examining within‐person fluctuation of arousal levels and engagement or preference of solitude throughout the day, we also expected that different people would gravitate toward solitude for arousal regulation to varying degrees. This is due in part because different personalities might be more prone to experiencing some types of affect more than others and might adopt different strategies (other than spending time alone) to regulate their experiences throughout the day. In the present research, we examined two specific dimensions of personality: extraversion–introversion dimension and neuroticism dimension. We were interested in whether people scoring higher on those personality traits might be more likely to seek solitude after experiencing highly aroused experiences.

#### 1.4.1 Neuroticism

Previous research shows some consistent patterns of how extraversion and neuroticism are linked to daily emotions, and particularly, physiological responses related to arousal dimension of emotions. Those high on neuroticism tend to experience more unpleasant mood states throughout the day (David et al., 1997; Wilt & Revelle, 2019). This is consistent with previous studies that show neuroticism correlates with experiencing more daily problems throughout the day as well as feeling more bothered and reactive to those problems (Barrett & Pietromonaco, 1997). Those high in neuroticism also experienced more instability of depressive mood throughout the day (Miller et al., 2009). In response to negative experiences which often elicit stronger physiological reactivity for those with higher neuroticism (Ormel et al., 2013), it has been suggested that these people are more likely to adopt disengaging coping strategies (Connor‐Smith & Flachsbart, 2007). Disengagement refers to behaviors such as trying to avoid or denying the problems, hiding emotions, or isolating oneself. Those responses do not necessarily mean that individuals with high neuroticism want to process and deal with the problems but rather to simply remove themselves from the sources of distress. As such, we predicted that high‐arousal negative mood would be more strongly linked to subsequent time spent in solitude for those higher in neuroticism.

#### 1.4.2 Extraversion–Introversion

On the other hand, extraversion consistently correlates with positive activation, either in general (Smillie et al., 2015) or throughout the day (Lucas et al., 2008). Extraverts are attracted to situations where they could experience particularly energizing states (Wilt & Revelle, 2019). The majority of those situations are inherently sociable, such as spending time with friends, or being at places with more opportunities for sociality, like at the gym, bars, and cafes (Matz & Harari, 2021). For this reason, it makes sense that extraverts have less desire to experience mood states that are low in activation (Rusting & Larsen, 1995, 1997), and thus less frequently spend time by themselves (Lucas et al., 2008). This suggests the opposite is true for introverts. It makes sense that, while extraverts are less prone and attracted to de‐activating emotions, introverts might be more likely to gravitate toward activities that allow them opportunities to downregulate especially after arousing experiences. Therefore, we expected introversion (low in extraversion) would strengthen the link between high‐arousal positive mood and subsequent time spent in solitude. For those high on extraversion, we predicted a nonlinear link between high‐arousal positive mood and subsequent time spent in solitude. A previous study showed that being extraverted is linked to delayed increase in fatigue 3 h later (Leikas & Ilmarinen, 2017), suggesting that extraverts might also need breaks after extended period of activation. So, for extraverts, perhaps likelihood of spending time alone is only heightened once high‐arousal positive mood reach certain levels. As such, we predicted that the moderation effect of extraversion–introversion on the link between high‐arousal positive affect with likelihood of being alone would be nonlinear.

## 2 METHODS

### 2.1 Recruitment

Four hundred participants signed up for our study that was advertised on multiple platforms: (1) the Durham University's Department of Psychology Research Participant pool hosted on SONA system, (2) the university's newsletters that are sent out to all staff and students, and (3) Prolific, Version 2023 (www.prolific.com). The study was advertised with the title “Study of daily behaviour and emotions,” and the description of the study made no mention of solitude being the targeted behavior. Originally, in Stage 1 manuscript, we only proposed to recruit from SONA participant pool and using the university's weekly newsletter. However, as recruitment slowed down and we did not achieve the planned sample size of 400, we revised our ethics application to be able to advertise the study on Academic Prolific.

### 2.2 Procedure

There were two parts to this study: one involved an initial questionnaire and the other involved hourly assessments of moods and activities throughout 1 day. The initial questionnaire was administered during an online induction session hosted on Qualtrics, Version 2023 (www.qualtrics.com). During this induction session, each participant was guided through instructions to download ExpiWell app on their smartphone (Version 2023). ExpiWell (www.expiwell.com) is an experience sampling application that can send signals to participants to complete EMA throughout the day. Once the participants downloaded ExpiWell, they saw two activities scheduled on the app. The first activity was an initial questionnaire that the participants took during the induction session, and the second activity consisted of 15 hourly surveys that were scheduled for the next day. As such, once participant downloaded the app, this would trigger the EMA surveys to be sent out the following day; this means, all participants started the next day for them whether it was a weekend or weekday.

#### 2.2.1 Initial questionnaire

##### Personality

We measured participants' levels of neuroticism and extraversion, using the McCrae and Costa's (1992) NEO‐PI‐R items from the International Personality Item Pool (Goldberg et al., 2006). The scales for Neuroticism and Extraversion–Introversion include 10 items each. The example items for Neuroticism are “often feel blue,” “am often down in the dumps,” or “am not easily bothered by things” (reverse). The example items for Extraversion–Introversion are “feel comfortable around people,” “am the life of the party,” or “have little to say” (reverse). Participants responded to each item on 5‐point scales, ranging from 1 = strongly disagree to 5 = strongly agree. Both scales yielded satisfactory internal consistency (Neuroticism: *ω*_*h*_ = 0.73; Extraversion: *ω*_*h*_ = 0.74).

##### Demographics

We asked participants to report their age, education, gender, ethnicity, socioeconomic status, and living arrangements. This demographic information was only collected for descriptive purposes so that we could assess the degree of generalizability of the findings.

#### 2.2.2 Ecological momentary assessments

After filling out the measures described above, participants received mobile notifications to fill out EMA the next day. EMA signals were scheduled every hour between 8 a.m. and 11 p.m. (maximum of 15 signals per participant, per day). Each survey took about 2 min to complete.

##### Emotions

To measure participants' current emotional arousal, we used items representing high‐arousal positive emotions (i.e., happy, delighted, excited, astonished, and aroused) and high‐arousal negative emotions (i.e., tense, alarmed, angry, afraid, annoyed, distressed, and frustrated). These items were selected from the list of items provided in Russell (1980; Table 2). Participants in the original study by Russell (1980) categorized these items into the Arousal dimension, with some representing positive states and other represented negative states. The ways that emotional states are categorized into the Arousal dimension of the circumplex were relatively consistent when tested in Estonian, Greek, and Polish (Russell et al., 1989). Therefore, these items appear to capture emotions that are high on arousal levels for the purpose of this research. Participants indicated on a scale from 0 and 10 to report how much of each emotion was experienced at the time of the signal, with 0 representing no experience whatsoever and 10 representing the most intense possible. Both measures of high‐arousal positive and high‐arousal negative emotions showed good reliabilities at both within (0.73 for positive and 0.82 for negative) and between (0.88 for positive and 0.95 for negative) levels.

##### Social vs. alone situations

To determine situations in which participants are alone, we asked the participants to report every hour about their current emotion and activity:

- [Alone 1:] What best describes your surrounding at the time you clicked on this survey? (Forced choice options: No one was around, I was with other people)
- [Alone 2:] What best describes the company you were with at that time? (Options—choose all that apply: stranger(s), family, friends, romantic partner, with a crowd). If “No one was around” is chosen for [Alone 1], the participants did not see this question and were directed to [Alone 3]
- [Alone 3:] At the time you clicked on this survey, have you been having a back‐and‐forth conversation with someone either remotely (on the phone), on the Internet, or in person? (Forced choice options: yes, no)

*Situation* was coded as 1 (for solitude) if participants choose the following combinations:

- If they choose “No one was around” for [Alone 1] AND “No” for [Alone 3]
- If they choose “strangers” OR “with a crowd” for [Alone 2] AND “No” for [Alone 3]

So, if participants reported being with friends or romantic partner but not interacting with them, such as reading books before bed or studying in the library together, they were not considered being alone. Similarly, if they reported that no one was around but they have been having a back‐and‐forth conversation with someone, presumably remotely or online (Campbell & Ross, 2021), they were not considered being alone. But, if participants reported being with strangers or with a crowd but not interacting with them, such as reading alone in a café or browsing alone in a busy shop, they were considered being alone. Therefore, this operationalization of solitude emphasizes the absence of interaction as the qualifier of this state. At the same time, it also excludes non‐interactive time spent with familiar others because we wanted to stay true to the definition of solitude that Long and Averill (2003) proposed “a state characterised by disengagement from the immediate demands of other people.” In other words, being near a familiar others might still introduce certain levels of social expectations and demands that might muddle the solitary state. This operationalization was determined at Stage 1 of this registered report.

After coding for the participant's current situation in each hourly assessment, we created a new variable to represent *transition* between social and solitary situations. This variable was determined by looking at situation reported an hour before at time (*t* – 1) and situation reported at time (*t*). As such, there were four possibilities that could happen. If a participant went from being in a social situation (coded as 0 for *situation*) to being in solitary situation (coded as 1 for *situation*), they got a combination of 0–1 (social‐to‐solitude). If the participant reported being in a social situation an hour before and continued in the same situation, they got a combination of 0–0 (social‐to‐social). Going from a solitary situation to a social situation resulted in a combination of 1–0 (solitude‐to‐social), and continuing from a solitary situation to another solitary situation resulted in a combination of 1–1 (solitude‐to‐solitude). *Transitions* from social‐to‐solitude were then coded as 1 and other *transitions* were coded as 0.

##### Preference

To measure participants' current preference for solitude versus being with others, we adapted from the measure used by Ren et al. (2016) and asked participants which of the following option is the most attractive to them at the current moment: “spend time alone,” “spend time with people,” “[if currently with people] spend time with someone else.” Preference to spend time alone was coded as 1, and other preferences were coded as 0.

#### 2.2.3 Schedule of measures

The figure below demonstrated how measures were scheduled. For each EMA, participants reported their current emotions and their situation in the past hour.

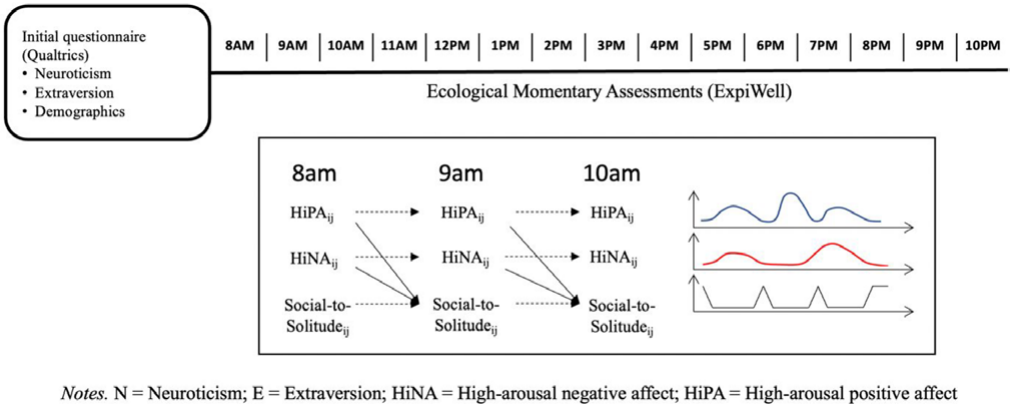

## 3 ANALYSIS PLAN

### 3.1 Variable standardization

High‐arousal positive affect and high‐arousal negative affect were standardized using person‐mean‐*SD* standardization approach (Wang et al., 2019). This standardization approach allowed us to assess the likelihood that someone would move from a social situation to solitude when their arousal levels increase 1 within‐person *SD* unit above their own mean. We also sample‐mean‐standardized neuroticism and extraversion scores. None of the other binary variables, including situations (solitary versus social), transitions (social‐to‐solitude transition versus other transitions), and preference (prefer solitude versus spending time with other), were standardized.

### 3.2 Hypotheses

The present research tested the hypotheses below.

#### 3.2.1 Hypothesis 1

Increases in high‐arousal negative affect reported at the previous signal (*t −* 1) would correlate positively with higher likelihood of transition from social‐to‐solitude reported at the subsequent signal (*t*), compared to other transitions (social‐to‐social, solitude‐to‐social, solitude‐to‐solitude). This positive correlation would be moderated by neuroticism, such that:

1.1 The correlation would be stronger at *higher* levels of neuroticism.
1.2 The correlation would be weaker at *lower* levels of neuroticism.

#### 3.2.2 Hypothesis 2

Increases in high‐arousal positive affect reported at the previous signal (*t −* 1) would correlate positively with higher likelihood of transition from social‐to‐solitude reported at the subsequent signal (*t*). This positive correlation would be moderated by extraversion, such that:

2.1 The correlation would be stronger at *lower* levels of extraversion.
2.2 The correlation would be weaker *higher* levels of extraversion.

#### 3.2.3 Hypothesis 3

Based on experimental findings that solitude leads to decreases in high‐arousal emotions, we also investigated whether these decreases would be moderated by extraversion and neuroticism levels. If low‐extraversion or high‐neuroticism people are more likely to seek solitude after experiencing high‐arousal emotions, they might show greater drops in arousal in solitude. As such, solitary *situation* would be associated with lower high‐arousal positive and negative affects compared to social *situation*. Since EMA were administered every 1 h, we did not expect a lag effect but only a main effect of *situation* (social versus solitary) at signal (*t*) on high‐arousal emotions at signal (*t*).

3.1 The difference between social versus solitary *situations* would be stronger at *higher* levels of neuroticism.
3.2 The difference between social versus solitary *situations* would be stronger at *lower* levels of extraversion.

### 3.3 Analyses and model selection process

To test each hypothesis, we used R (RStudio Team, 2023) and performed the original models proposed in Stage 1 manuscript. If we saw singularity or non‐convergence warnings, we examined the outputs to see whether there was multi‐collinearity and revised or simplified the model. Model selection processes were detailed in the .Rmd file including in this paper's Open Science Framework page: https://osf.io/cd63x/?view_only=dd3fce6409af4d6797942e02a914b475.

To test Hypotheses 1 and 2, we conducted mixed‐effects logistic regression using “*glmer*” function in the “*lmer4*” R package (version 1.1–30; Bates et al., 2015). In Stage 1 proposal, we planned to put *transition* at a subsequent data point (*t*) as outcome variable *and* regress this variable on linear and quadratic terms of *time* (*time and time^2*), high‐arousal negative/positive affect at (*t* – 1), neuroticism/extraversion, and the interaction term of affect and personality. We included random intercept and random slopes for the *time* variables (both linear and quadratic components). We included quadratic term of *time* to capture any nonlinear change in outcome variables but we only hypothesized linear changes. Because the outcome was binomial, we used “family = binomial(‘logit’)” argument and maximized the models using “bobyqa.”

We deviated from the originally planned models for both Hypothesis 1 and 2 in two ways. First, we replaced the outcome variable because the *transition* variable was problematic. As a reminder of the original plan, we created this variable by assigning 1 value to only the transition from social situation at (*t* – 1) to solitude at (*t*) whereas other transitions were coded as 0. However, after going from a social situation to solitude, it is only possible for a person to either go from solitude back to social situation or to remain in solitude; as such, this necessarily ruled out the possibility of a social‐to‐social transition. As such, all levels of the *transition* variable did not have equal probabilities. To fix this problem, instead of using the *transition* variable as outcome, we used current reported situation as outcome and controlled for reported situation at the preceding time point in the models. The second deviation from the originally planned models was that we replaced *time* and *time^2* with linear and quadratic *time* components that were orthogonal (using *poly()* function); that is, they were independently scaled and centered to avoid correlation between them. Once these two changes were made, if the revised models continued to show either non‐convergence or singularity warnings, we simplified the models following the two steps; first, remove the fixed effect of quadratic *time* component, and second, remove the random slope of linear *time* component.

To test exploratory Hypothesis 3, we conducted linear mixed‐effects models using “*lmer*” and regressed high‐arousal positive and negative affect measured at time (*t*) onto linear and quadratic terms of time, and *situation* (0 = social, 1 = solitude) measured at the same time point. Again, we used linear and quadratic *time* components that were orthogonal (using *poly()* function) and included random intercepts as well as random slopes for both linear and quadratic components in the models.

### 3.4 Stage 1 registered report power analysis

Prior to data collection, we performed data simulation to test the proposed models using R (version 2022.02.0). If 200 out of 400 participants fill out all 15 EMAs, that means a total of 3000 observations. Because the outcome variable (e.g., *solitude* vs. *social situation*) was lagged 1 time point and its first report of the day would not be counted, only 2800 would be included in the planned analyses. Power simulation with 100 repetitions of the above models using the “simr” package (version 1.0.5) suggests that we should have more than 80% power to detect a fixed effect as small as 0.20 for the interaction term in each model.

### 3.5 Sample

Out of 400 participants that clicked on our survey to consent their participation, 391 downloaded the ExpiWell app (see more details in Procedure). Of these participants, we excluded a total of 29 participants due to the following reasons: (1) seven participants were duplicates, (2) two participants were under 18, which violated one of our inclusion criteria that participants must be 18 or above, (3) five participants either filled out more than 1 day of surveys or submitted more than 15 surveys, (4) nine did not complete the initial questionnaire, and (5) six did not complete any later EMAs.

The final sample had 362 participants between the ages of 18 and 66 (*M*_age_ = 26.22, *SD*_age_ = 9.56; one participant did not report age). There were 263 female and 91 male participants (seven identified as non‐binary and one preferred not to report sex). We reported other demographic characteristics of the sample in Table 1.

**TABLE 1:** Demographics.

	*N*	%
*Ethnicity* (* recoded from open‐ended responses)		
White	256	70.72%
Black	7	1.93%
South Asian (Indian, Pakistan, etc.)	26	7.18%
Central Asian	5	1.38%
East Asian (Chinese, Japanese, etc.)	52	14.36%
Southeast Asian*	7	1.93%
Indigenous	0	0.00%
Prefer not to say	4	1.10%
Mixed race*	3	0.83%
Hispanic/Latino*	2	0.55%
*Employment status*		
Full time	95	26.24%
Part time	38	10.50%
Student	206	56.91%
Stay at home parent	6	1.66%
Retired	2	0.55%
Unemployed and looking	8	2.21%
Other	7	1.93%
*Marital status*		
Married	53	14.64%
Unmarried	297	82.04%
Separated	3	0.83%
Divorced	8	2.21%
Widowed	1	0.28%
*Highest education*		
Some secondary education or less	4	1.10%
Diploma from secondary education or equivalent (high school diploma, GED, GCSE, etc.)	87	24.03%
Some college, but no degree	127	35.08%
Associates or technical degree	6	1.66%
Bachelor's degree	78	21.55%
Graduate or professional degree (MA, MS, MBA, PhD, JD, MD, DDS, etc.)	54	14.92%
Prefer not to say	6	1.66%
*Household size*		
1 person	57	15.75%
2 persons	66	18.23%
3 persons	66	18.23%
4 persons	75	20.72%
More than 4 persons	98	27.07%

In total, we collected 4338 hourly surveys from 362 participants. Only 103 participants (28.45%) completed all 15 hourly surveys, which is significantly lower than what we had expected. The rest of the participants completed decent numbers of survey, with 172 participants (47.51%) submitting between 11 and 14 surveys, 58 (16.02%) completing between 6 and 10 surveys, and 29 (8.01%) completing between 1 and 5 surveys.

## 4 RESULTS

### 4.1 Missing data analyses

Table 2 showed correlations between personality variables (i.e., neuroticism and extraversion), total number of surveys each person submitted, the proportion of surveys submitted on weekends, the proportion of surveys submitted that was reported when the person was alone vs. with others, or preferred being alone vs. with others, and the average levels of high‐arousal negative and positive emotions the person experienced that day. There were no significant correlations between neuroticism and extraversion with the numbers of survey submitted. So, we could conclude that missing data were not a function of individuals' personality characteristics. Overall, 97 participants completed EMA surveys over the weekends, but whether participants completed surveys on a weekday or a weekend did not show significant correlations with any of the other main variables.

**TABLE 2:** Correlations between main variables (mean scores calculated within person).

	*M*	*SD*	1	2	3	4	5	6	7
1. Neuroticism	2.95	0.70							
2. Extraversion	3.16	0.72	−0.46** [−0.53, −0.37]						
3. Number of surveys submitted	11.98	3.44	−0.06 [−0.17, 0.04]	−0.01 [−0.11, 0.10]					
4. Surveys completed on weekends	0.27	0.44	−0.06 [−0.16, 0.04]	0.04 [−0.06, 0.15]	−0.07 [−0.17, 0.04]				
5. Percentage of solitude vs. social situation (%)	48.46	25.20	0.07 [−0.03, 0.17]	−0.09 [−0.19, 0.01]	−0.15** [−0.25, −0.05]	−0.03 [−0.14, 0.07]			
6. Preference for solitude vs. social situation (%)	52.63	28.68	0.14** [0.03, 0.24]	−0.24** [−0.34, −0.14]	−0.07 [−0.18, 0.03]	−0.10 [−0.20, 0.01]	0.54** [0.46, 0.61]		
7. High‐arousal negative emotions	2.45	1.25	0.31** [0.21, 0.40]	−0.12* [−0.22, −0.02]	−0.14** [−0.24, −0.04]	−0.02 [−0.13, 0.08]	0.14** [0.04, 0.24]	0.18** [0.08, 0.28]	
8. High‐arousal positive emotions	3.30	1.23	−0.26** [−0.35, −0.16]	0.22** [0.12, 0.31]	−0.00 [−0.10, 0.10]	0.01 [−0.09, 0.12]	−0.1** [−0.25, −0.05]	−0.27** [−0.36, −0.17]	−0.05** [−0.08, −0.02]

*Note*: *M* and *SD* are used to represent mean and standard deviation, respectively. Values in square brackets indicate the 95% confidence interval. The confidence interval is a plausible range of population correlations that could have caused the sample correlation (Cumming, 2014).

* *p* < 0.05.

** *p* < 0.01.

People who submitted more surveys were less likely to report being alone in those surveys (*r* = −0.15, *p* < 0.01) and on average experienced lower high‐arousal negative emotions (*r =* −0.14, *p* < 0.01). There was also a small positive correlation between average levels of high‐arousal negative emotions and likelihood of being alone while filling out surveys (*r* = 0.14, *p* < 0.01). We performed a regression analysis to investigate whether participants who submitted more surveys and had an overall unhappy day would submit more surveys when they were alone, and we did not find evidence that this was the case. There was no interaction between number of surveys submitted and high‐arousal negative emotions predicting proportion of solitude situations reported (see Supplementary Materials; Table SM1). This means that we did not over‐sample alone situations on unhappy days. However, there might be a problem (although very minor because the correlations were small) with under‐sampling alone situations in general because participants who filled out more surveys were more likely to report being with others. We also appeared to sample more surveys on days when people were happier.

### 4.2 Preliminary analyses

#### 4.2.1 Do people usually prefer being alone when they are alone?

The results showed that whether a person was currently alone or with others had a significant positive effect on their preference (*b* = 2.37, *SE* = 0.11, *z* = 22.52, *p* < 0.001). That means, for each unit increase in the person's current situation (i.e., currently alone), the odds of preferring solitude are approximately 10.7 times more likely (exp(2.37) = 10.70). In other words, if a person was alone at the time they filled out the survey, there was 92% probability that they would prefer being alone at that time (10.7/(1 + 10.7) = 0.915) (see Supplementary Materials; Table SM2).

#### 4.2.2 How does high‐arousal emotions change over the course of a day?

We found a significant interaction of quadratic effect of time with situation on high‐arousal positive emotions (*b* = −4.04, *SE* = 1.98, *t* = −2.04, *p* = 0.041). On average for each person, social situation was associated with significantly more high‐arousal positive emotions than alone situation (*b* = −0.21, *SE* = 0.03, *t* = −7.44, *p* < 0.001). This difference became larger in the afternoon and was driven by the rise in high‐arousal positive emotions in social situations around 4 PM. There was no quadratic effect of time nor interaction between time and situation on high‐arousal negative emotion. Instead, there was a significant linear decrease in high‐arousal negative emotions for both social and alone situation as time goes on throughout the day (*b* = −0.13, *SE* = 1.68, *t* = −7.60, *p* < 0.001) (see Supplementary Materials; Table SM3 and Figure SM4).

#### 4.2.3 Do people's likelihood of being alone versus with others, and their preference, change over the course of a day?

On average, our participants reported being alone 48.46% of the times that they submitted surveys, and preferring to be alone 52.63% of the times (see Table 2). To test whether people on average preferred being alone more often than they were spending time alone, we performed a one‐sample weighted *t*‐test comparing the two variables and weighted by the numbers of surveys people completed. We found that people on average preferred being alone more often than they were actually alone (*t*(361) = 3.39, *p* < 0.001).

Likelihood of someone's being alone and preferring to be alone appeared to change throughout the day. We found that as the day progressed, the likelihood of someone reporting being alone decreased linearly between the morning and the afternoon and remained relatively at the same levels until the evening. This was evidenced by a significant quadratic effect of time on the situation variable (*b* = 13.04, *SD* = 3.21, *z* = 4.06, *p* < 0.001). Controlling for the situation that people reported being in (since a person who was alone was likely to report preferring being alone), preference for solitude also decreased linearly between the morning and the afternoon, remained relatively at the same levels in the afternoon hours, but appeared to increase again in the evening. Again, this was evidenced by a significant quadratic effect of time on the preference variable (*b* = 14, 09, *SD* = 2.74, *z* = 5.15, *p* < 0.001). These findings suggest that, for this sample, afternoons and evenings were the times when people tended to become more socially active; however, preference for solitude began to rise toward the evenings (see Figure SM5).

### 4.3 Hypothesis testing

#### 4.3.1 Hypothesis 1

##### Confirmatory analyses

The results did not support our hypothesis. We did not find the main effect of high‐arousal negative emotions predicting being alone at a later time point, nor the interaction between high‐arousal negative emotions and neuroticism (see Table 3). We found that, if a participant reported being alone at the previous time point, there was a 75% probability that they would be alone at a later point (*b* = 1.12, *SE* = 0.09, *z* = 12.22, *p* < 0.001).

**TABLE 3:** Results of mixed‐effects logistic regression testing the interaction of neuroticism and high‐arousal negative emotions an hour before predicting likelihood of solitude versus social situation 1 h later.

	*b*	*SE*	*z*	*p*
(intercept)	−0.47	0.11	−4.10	<0.001
*Within‐person effects*				
Time	−0.04	0.01	−3.65	< 0.001
Situation at (*t* – 1)	1.12	0.09	12.23	<0.001
High‐arousal negative emotions at (*t* – 1)	−0.03	0.04	−0.79	0.430
*Between‐person effect*				
Neuroticism (mean‐centered)	0.08	0.08	1.04	0.297
*Interaction*				
Neuroticism × High‐arousal negative emotions at (*t* – 1)	−0.01	0.06	−0.20	0.845

*Note*: This is a simplified model that deviates from the proposed analysis in Stage 1 Registered Report. Current situation is used as outcome variable (solitude vs. social situation). The model includes the following fixed effects: linear term of time, lagged variable of situation and high‐arousal negative emotions at the previous time point (*t* – 1), mean‐centered neuroticism, and the interaction of neuroticism and high‐arousal negative emotions. Random effects include random intercept and random slope of time. The model uses “family = binomial(‘logit’)” with “bobyqa” maximizer.

##### Exploratory analyses

We also performed a model predicting participants' preferred situation with their current levels of high‐arousal negative emotions and the interaction between high‐arousal negative emotions with neuroticism. We controlled for participants' preferred situation an hour before. We found main effects of both high‐arousal negative emotions and neuroticism. Participants higher on neuroticism were more likely to prefer being alone (*b* = 0.26, *SE* = 0.08, *z* = 3.26, *p* = 0.001). Experiencing high‐arousal negative emotions predicted higher preference for solitude (*b* = 0.27, *SE* = 0.04, *z* = 6.21, *p* < 0.001), but this was not moderated by neuroticism levels (see Table 4).

**TABLE 4:** Results of mixed‐effects logistic regression testing the interaction of neuroticism and current high‐arousal negative emotions predicting current preference for solitude versus social situation.

	*b*	*SE*	*z*	*p*
(intercept)	−0.72	0.12	−5.86	<0.001
*Within‐person effects*				
Survey	−0.02	0.01	−1.57	0.118
Preference at (*t* – 1)	1.72	0.09	18.37	<0.001
High‐arousal negative emotions at (*t*)	0.27	0.04	6.21	<0.001
*Between‐person effect*				
Neuroticism (mean‐centered)	0.26	0.08	3.26	0.001
*Interaction*				
Neuroticism × High‐arousal negative emotions at (*t*)	−0.03	0.07	−0.49	0.622

*Note*: In this model, current preference for solitude vs. social situation is used as outcome variable. The model includes the following fixed effects: linear term of time, lagged variable of preference the previous time point (*t* – 1), high‐arousal negative emotions at present time (*t*), mean‐centered neuroticism, and the interaction of neuroticism and high‐arousal negative emotions. Random effects include random intercept only because model that includes random slope did not converge. The model uses “family = binomial(‘logit’)” with “bobyqa” maximizer.

**FIGURE 1:**
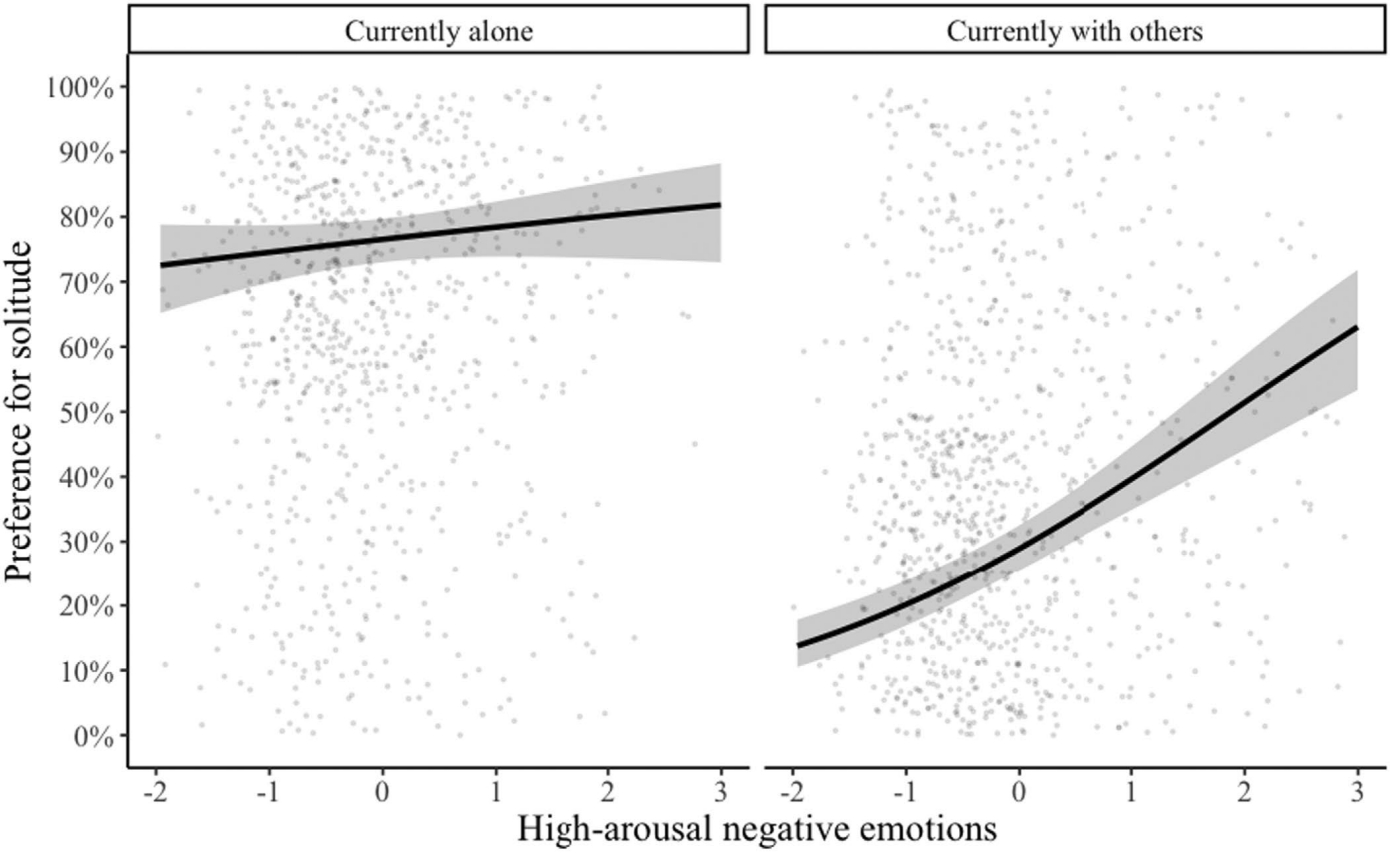
Graph showing the interaction of current situation and current high‐arousal negative emotions predicting current preference for solitude versus social situation (Table 5).

We conducted additional analyses to investigate if high‐arousal negative emotions predict the preference for solitude differently depending on the participant's current situation. It may be the case that experiencing high‐arousal negative emotions makes people want to change their current situation. If so, we should expect that people would prefer solitude more when high‐arousal negative emotions were experienced with other people, but they would prefer social interactions more if those emotions were experienced when they were alone. We found a significant interaction between high‐arousal negative emotions with the participants' current situation (*b* = −0.37, *SE* = 0.10, *z* = −3.66, *p* < 0.001) (see Table 5). The more high‐arousal negative emotions that a person was experiencing at the current moment when they were in social interaction, the higher their preference for solitude (see Figure 1). However, if they experienced high‐arousal negative emotions when they were alone, they continued to prefer being alone, instead of wanting to be with other people.

**TABLE 5:** Results of mixed‐effects logistic regression testing the interaction of current situation and current high‐arousal negative emotions predicting current preference for solitude versus social situation.

	*b*	*SE*	*z*	*p*
(intercept)	−1.54	0.16	−9.81	<0.001
*Within‐person effects*				
Survey	−0.01	0.01	−0.71	0.477
Preference at (*t* – 1)	1.39	0.11	12.65	<0.001
Situation at (*t*)	2.08	0.11	18.46	<0.001
High‐arousal negative emotions at (*t*)	0.48	0.06	7.53	<0.001
*Interaction*				
Situation × High‐arousal negative emotions at (*t*)	−0.37	0.10	−3.66	<0.001

*Note*: In this model, current preference for solitude vs. social situation is used as outcome variable. The model includes the following fixed effects: linear term of time, lagged variable of preference the previous time point (*t* – 1), current situation at time (*t*), current high‐arousal negative emotions at time (*t*), and the interaction of situation and high‐arousal negative emotions. Random effects include random intercept and random slope of time. The model uses “family = binomial(‘logit’)” with “bobyqa” maximizer.

**FIGURE 2:**
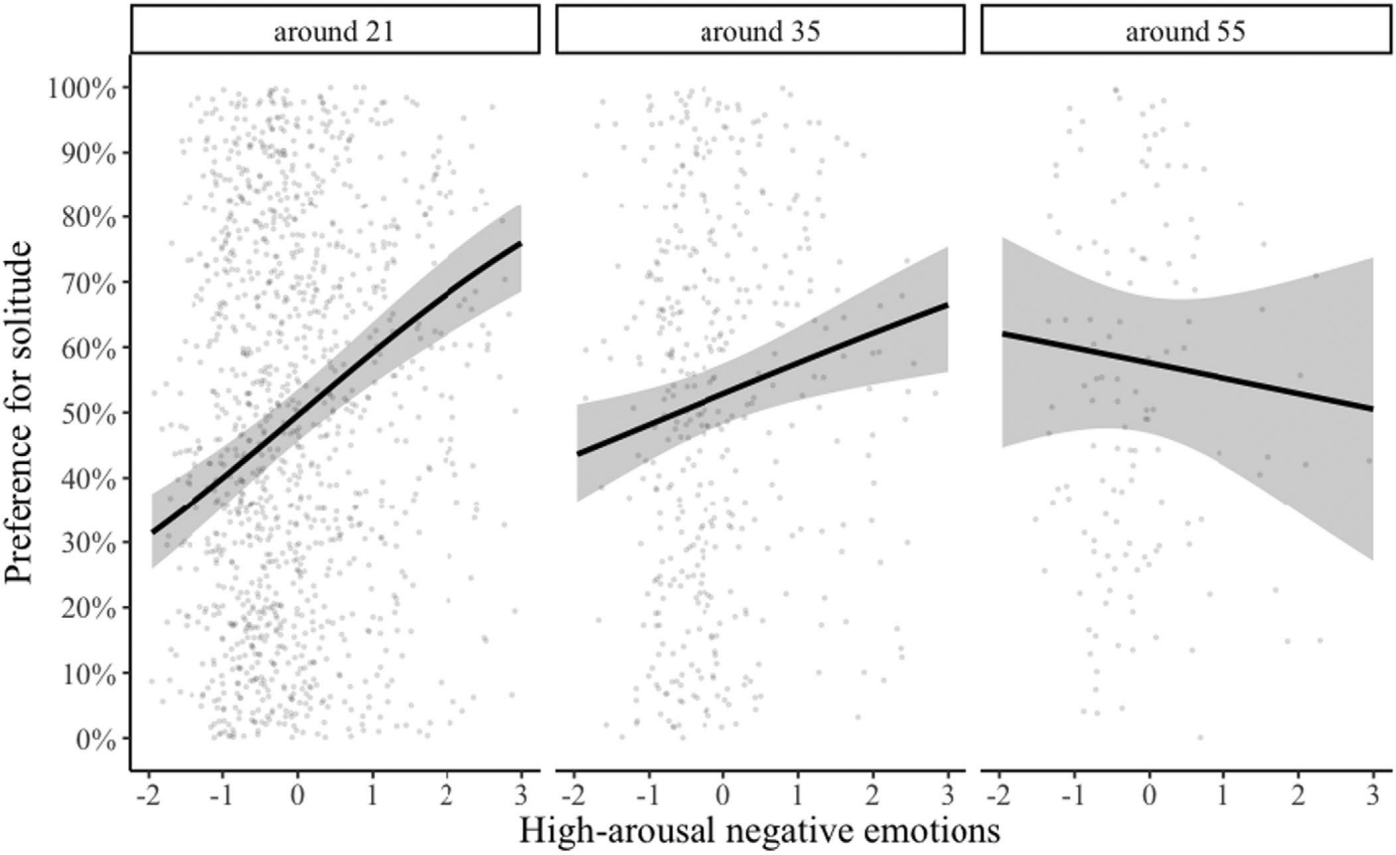
Graph showing the interaction of current situation, current high‐arousal negative emotions, and age predicting current preference for solitude versus social situation (Table 6). Here, we specified *interact_plot* (in R package *interactions*) to give us slopes for values of 21, 35, and 55 on age variable.

Based on previous findings by Pauly et al. (2017), we entered participants' age as an additional moderator. We found that the association between high‐arousal negative emotions and preference for solitude was moderated by age (*b* = −0.01, *SE* = 0.01, *z* = −2.37, *p* = 0.018) (see Table 6). High‐arousal negative emotions were more strongly linked to preference for solitude for younger participants and became weaker as age increases (see Figure 2).

**TABLE 6:** Results of mixed‐effects logistic regression testing the interaction of current situation, current high‐arousal negative emotions, and age predicting current preference for solitude versus social situation.

	*b*	*SE*	*z*	*p*
(intercept)	−1.54	0.16	−9.72	<0.001
*Within‐person effects*				
Survey	−0.01	0.01	−0.76	0.450
Preference at (*t* – 1)	1.39	0.11	12.57	<0.001
Situation at (*t*)	2.07	0.11	18.38	<0.001
High‐arousal negative emotions at (*t*)	0.49	0.06	7.62	<0.001
*Between‐person effect*				
Age (mean‐centered)	0.01	0.01	1.70	0.089
Interactions				
Situation × High‐arousal negative emotions at (*t*)	−0.39	0.10	−3.80	<0.001
Situation × Age	−0.01	0.01	−1.04	0.298
High‐arousal negative emotions × Age	−0.01	0.01	−2.37	0.018
Situation × High‐arousal negative emotions × Age	−0.00	0.01	−0.08	0.933

*Note*: In this model, current preference for solitude vs. social situation is used as outcome variable. The model includes the following fixed effects: linear term of time, lagged variable of preference the previous time point (*t* – 1), current situation at time (*t*), current high‐arousal negative emotions at time (*t*), age, and all two‐way and three‐way interactions of situation, high‐arousal negative emotions, and age. Random effects include random intercept and random slope of time. The model uses “family = binomial(‘logit’)” with “bobyqa” maximizer.

#### 4.3.2 Hypothesis 2

##### Confirmatory analyses

Hypothesis 2 was also not supported. We did not find the main effect of high‐arousal positive emotions predicting being alone at a later time point nor the interaction with extraversion (see Table 7).

**TABLE 7:** Results of mixed‐effects logistic regression testing the interaction of extraversion and high‐arousal positive emotions an hour before predicting likelihood of solitude versus social situation 1 h later.

	*b*	*SE*	*z*	*p*
(intercept)	−0.50	0.11	−4.40	<0.001
*Within‐person effects*				
Survey	−0.04	0.01	−3.60	< 0.001
Situation at (*t*– 1)	1.14	0.09	13.06	<0.001
High‐arousal positive emotions at (*t* – 1)	−0.02	0.04	−0.47	0.639
*Between‐person effect*				
Extraversion	−0.07	0.07	−0.96	0.340
*Interaction*				
Extraversion × High‐arousal positive emotions at (*t* – 1)	0.06	0.05	1.03	0.302

*Note*: This is a simplified model that deviates from the proposed analysis in Stage 1 Registered Report. Current situation is used as outcome variable (solitude vs. social situation). The model includes the following fixed effects: linear term of time, lagged variable of situation and high‐arousal positive emotions at the previous time point (*t* – 1), mean‐centered extraversion, and the interaction of extraversion and high‐arousal negative emotions. Random effects include random intercept only because the model with random slope of time yields singular fit. The model uses “family = binomial(‘logit’)” with “bobyqa” maximizer.

##### Exploratory analyses

Instead, for the preference outcome, the model showed main effects of both high‐arousal positive emotions and extraversion. Participants higher on extraversion were less likely to prefer being alone (*b* = −0.43, *SE* = 0.09, *z* = −4.79, *p* < 0.001). Opposite to what we expected, experiencing high‐arousal positive emotions predicted lower preference for being alone at the same time point (*b* = −0.53, *SE* = 0.05, *z* = −11.27, *p* < 0.001), but this was not moderated by extraversion levels (see Table 8).

**TABLE 8:** Results of mixed‐effects logistic regression testing the interaction of extraversion and current high‐arousal positive emotions predicting current preference for solitude versus social situation.

	*b*	*SE*	*z*	*p*
(intercept)	−0.55	0.14	−3.92	<0.001
*Within‐person effects*				
Survey	−0.03	0.01	−2.20	0.028
Preference at (*t* – 1)	1.53	0.10	14.62	<0.001
High‐arousal positive emotions at (*t*)	−0.53	0.05	−11.27	<0.001
*Between‐person effect*				
Extraversion	−0.43	0.09	−4.79	<0.001
*Interaction*				
Extraversion **×** High‐arousal positive emotions at (*t* – 1)	−0.04	0.07	−0.64	0.522

*Note*: In this model, current preference for solitude vs. social situation is used as outcome variable. The model includes the following fixed effects: linear term of time, lagged variable of preference the previous time point (*t* – 1), high‐arousal positive emotions at present time (*t*), mean‐centered extraversion, and the interaction of extraversion and high‐arousal positive emotions. Random effects include random intercept and random slope of time. The model uses “family = binomial(‘logit’)” with “bobyqa” maximizer.

**FIGURE 3:**
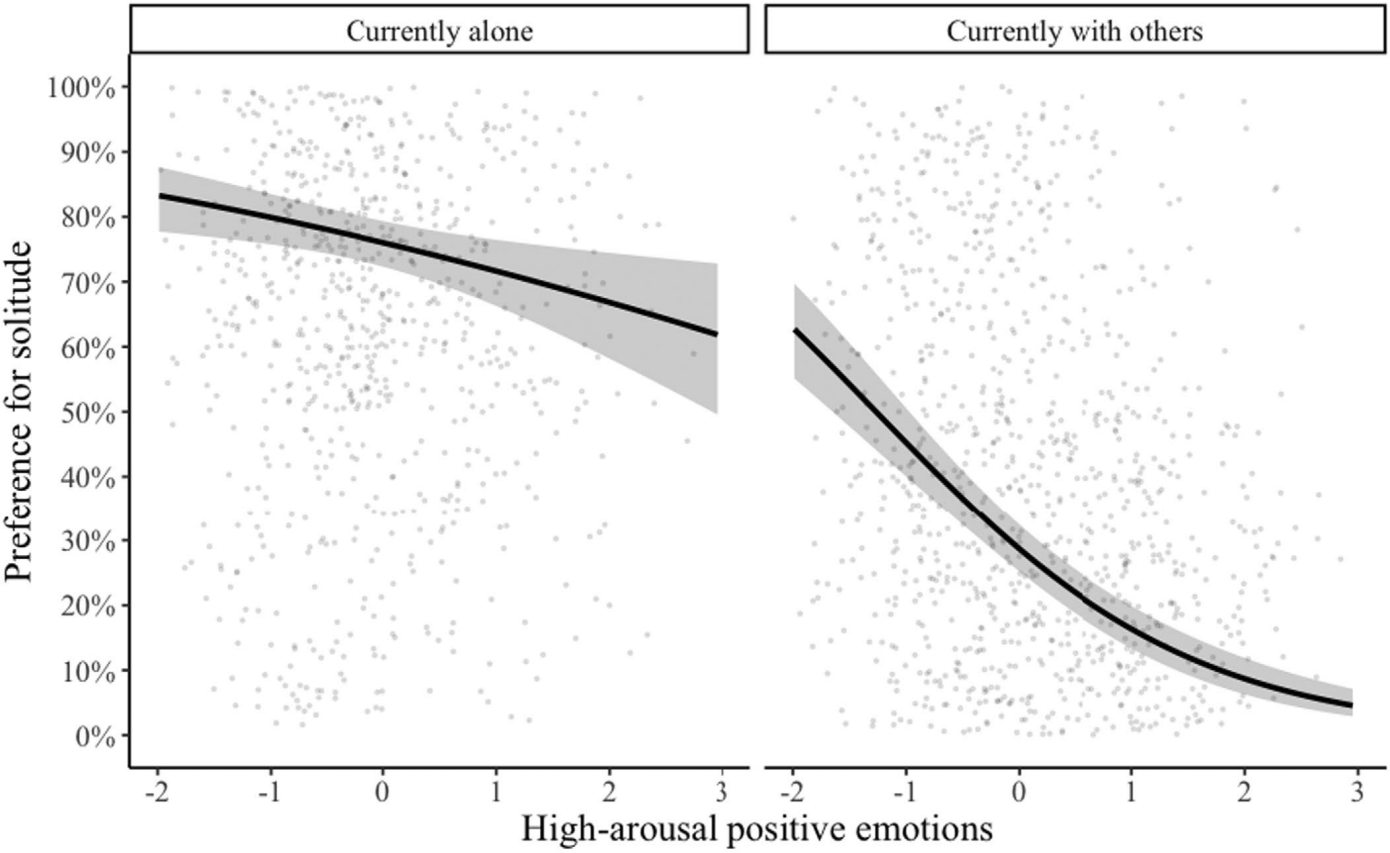
Graph showing the interaction of current situation and current high‐arousal positive emotions predicting current preference for solitude versus social situation (Table 9).

We found a significant interaction between high‐arousal positive emotions with the participants' current situation predicting preference for solitude (*b* = 0.49, *SE* = 0.10, *z* = 4.66, *p* < 0.001) (see Table 9). Preference for solitude decreased when high‐arousal positive emotions were heightened during solitude and social situations, and the decrease was more pronounced when high‐arousal positive emotions were experienced in social situations (see Figure 3). We did not find that age moderated the effect of high‐arousal positive emotions on preference (see Table 10).

**TABLE 9:** Results of mixed‐effects logistic regression testing the interaction of current situation and current high‐arousal positive emotions predicting current preference for solitude versus social situation.

	*b*	*SE*	*z*	*p*
(intercept)	−1.48	0.16	−9.14	<0.001
*Within‐person effects*				
Survey	−0.01	0.01	−1.06	0.291
Preference at (*t* – 1)	1.32	0.11	11.80	<0.001
Situation at (*t*)	2.06	0.11	18.10	<0.001
High‐arousal positive emotions at (*t*)	−0.72	0.07	−10.03	<0.001
*Interaction*				
Situation × High‐arousal positive emotions at (*t*)	0.49	0.10	4.66	<0.001

*Note*: In this model, current preference for solitude vs. social situation is used as outcome variable. The model includes the following fixed effects: linear term of time, lagged variable of preference the previous time point (*t* – 1), current situation at time (*t*), current high‐arousal positive emotions at time (*t*), and the interaction of situation and high‐arousal positive emotions. Random effects include random intercept and random slope of time. The model uses “family = binomial(‘logit’)” with “bobyqa” maximizer.

**TABLE 10:** Results of mixed‐effects logistic regression testing the interaction of current situation, current high‐arousal positive emotions, and age predicting current preference for solitude versus social situation.

	*b*	*SE*	*z*	*p*
(intercept)	−1.54	0.15	−10.42	<0.001
*Within‐person effects*				
Survey	−0.01	0.01	−0.69	0.490
Preference at (*t* – 1)	1.41	0.10	13.73	<0.001
Situation at (*t*)	1.98	0.10	18.87	<0.001
High‐arousal positive emotions at (*t*)	−0.70	0.07	−10.12	<0.001
*Between‐person effect*				
Age	0.01	0.01	1.40	0.161
*Interactions*				
Situation **×** High‐arousal positive emotions at (*t*)	0.48	0.10	4.71	<0.001
Situation × Age	−0.00	0.01	−0.47	0.637
High‐arousal positive emotions **×** Age	0.01	0.01	1.12	0.265
Situation **×** High‐arousal positive emotions **×** Age	−0.00	0.01	−0.17	0.865

*Note*: In this model, current preference for solitude vs. social situation is used as outcome variable. The model includes the following fixed effects: linear term of time, lagged variable of preference the previous time point (*t* – 1), current situation at time (*t*), current high‐arousal positive emotions at time (*t*), age, and all two‐way and three‐way interactions of situation, high‐arousal positive emotions, and age. Random effects include random intercept only because model that also includes random slope did not converge. The model uses “family = binomial(‘logit’)” with “bobyqa” maximizer.

#### 4.3.3 Hypothesis 3

We tested whether being alone would be associated with lower high‐arousal negative and positive emotions, and whether this effect would be moderated by neuroticism or extraversion. We did not find a main effect of being alone versus with others on high‐arousal negative emotions, and there was no interaction with neuroticism. We found a main effect of being alone on high‐arousal positive emotions (*b* = −0.21, *SE* = 0.02, *t* = −7.52, *p* < 0.001), but this effect was not moderated by extraversion (see Table 11).

**TABLE 11:** Results of linear mixed‐effects regression testing the interaction of current situation, personality traits (neuroticism or extraversion), predicting current emotional states (negative or positive emotions).

	Predicting high‐arousal negative emotions				Predicting high‐arousal positive emotions			
	*b*	*SE*	*t*	*p*	*b*	*SE*	*t*	*p*
(intercept)	0.33	0.04	7.57	<0.001	−0.01	0.05	−0.25	0.807
*Within‐person effects*								
Survey	−0.04	0.00	−9.01	<0.001	0.01	0.01	2.69	0.008
Situation at (*t*)	0.02	0.03	0.55	0.581	−0.21	0.03	−7.52	<0.001
*Between‐person effects*								
Neuroticism	−0.01	0.03	−0.34	0.731				
Extraversion					−0.02	0.03	−0.65	0.517
*Interaction*								
Neuroticism **×** Situation at (*t*)	0.01	0.04	0.35	0.726				
Extraversion × Situation at (*t*)					0.03	0.04	0.89	0.375

*Note*: Two separate models are presented here side by side, one predicting negative emotions in the left and one predicting positive emotion in the right. Each model includes the following fixed effects: linear term of time, current situation at time (*t*), mean‐centered personality trait (neuroticism for negative emotion model and extraversion for positive emotion model), and the interaction between personality trait and current situation. Random effects include random intercept and random slope of time. Maximum likelihood estimations are used.

**FIGURE 4:**
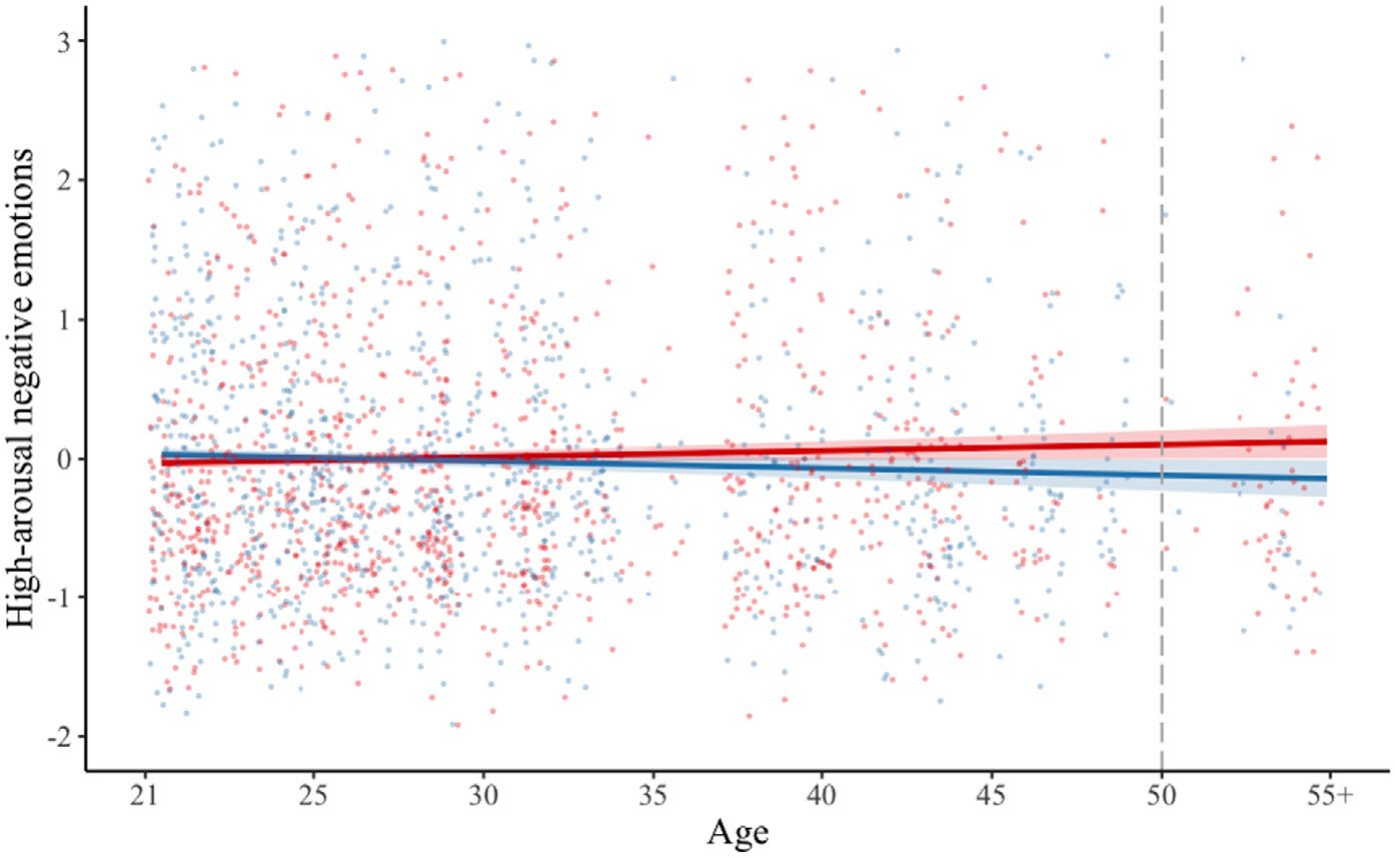
Graph showing the interaction of current situation and age, predicting current high‐arousal negative emotions (Table 12). Red line represents social situation; blue line represents solitude situation; dashed gray line represents value on the moderator (age) where there is a significant difference between solitude versus social situation on high‐arousal negative emotions.

**FIGURE 5:**
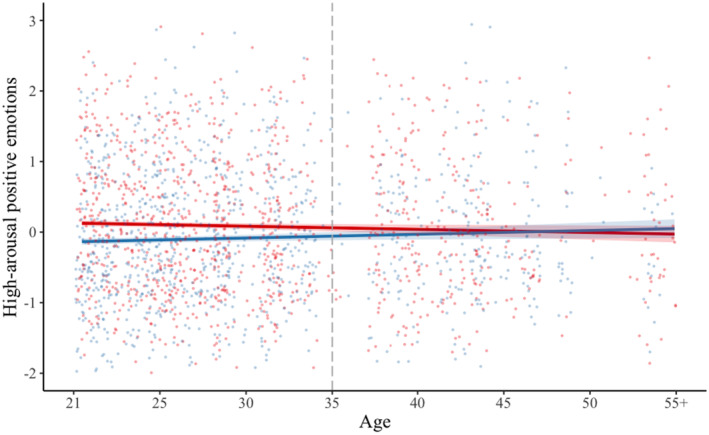
Graph showing the interaction of current situation and age, predicting current high‐arousal positive emotions (Table 12). Red line represents social situation; blue line represents solitude situation; dashed gray line represents value on the moderator (age) where there is a significant difference between solitude versus social situation on high‐arousal positive emotions.

We found a significant interaction between solitude versus social situation with age for both high‐arousal negative emotions (*b* = −0.01, *SE* = 0.002, *t* = −3.24, *p* = 0.001) and high‐arousal positive emotions (*b* = −0.01, *SE* = 0.003, *t* = 3.36, *p* < 0.001) (see Table 12). Investigations of marginal means suggested that the differences between solitude versus social situations on high‐arousal negative emotions were not significant for younger ages but became significant for those above 50 years old. On the other hand, the differences between situations on high‐arousal positive emotions were significant for younger age groups but not for those around 35 years old or above. As such, in this sample, being alone was associated with lower high‐arousal positive emotions for younger participants and lower high‐arousal negative emotions for older participants (see Figures 4 and 5). The above findings were not changed when we controlled for high‐arousal emotions reported at the previous time point.

**TABLE 12:** Results of linear mixed‐effects regression testing the interaction of current situation and age, predicting current emotional states (negative or positive emotions).

	Predicting high‐arousal negative states				Predicting high‐arousal positive states			
	*b*	*SE*	*t*	*p*	*b*	*SE*	*t*	*p*
(intercept)	0.33	0.04	7.54	<0.001	−0.01	0.05	−0.18	0.856
*Within‐person effects*								
Survey	−0.04	0.00	−8.97	<0.001	0.01	0.01	2.65	0.008
Situation at (*t*)	0.17	0.03	0.59	0.555	−0.22	0.03	−7.62	<0.001
*Between‐person effects*								
Age	0.04	0.00	2.30	0.022	−0.00	0.00	−2.33	0.020
*Interaction*								
Age × Situation at (*t*)	−0.01	0.00	−3.24	0.001	0.01	0.00	3.36	<0.001

*Note*: Two separate models are presented here side by side, one predicting negative emotions in the left and one predicting positive emotion in the right. Each model includes the following fixed effects: linear term of time, current situation at time (*t*), mean‐centered age, and the interaction between age and current situation. Random effects include random intercept and random slope of time. Maximum likelihood estimations are used.

## 5 DISCUSSION

This research explored arousal levels as a driving factor for solitude‐seeking, based on the premise that solitude downregulates high‐arousal emotions (Nguyen et al., 2018). We hypothesized that solitude becomes more likely and desirable when high‐arousal emotions are heightened. To investigate this, we tracked individuals' hourly experiences between 8 AM and 10 PM. We found that occurrence of solitude and preference for it changed throughout the day. As the day progressed, incidents of solitude decreased linearly between the morning and the afternoon and remained relatively at the same level until the evening. Controlling for the situation that people reported being in (since a person who was alone was likely to report preferring being alone) at the time of the signal, preference for solitude also decreased linearly between the morning and the afternoon, remained relatively at the same level in the afternoon hours, but appeared to increase again in the evening.

Contrary to our hypotheses, lagged analyses did not indicate high‐arousal emotions predicting being alone an hour later. However, individuals were more likely to prefer solitude while experiencing high‐arousal negative emotions, and less so while experiencing high‐arousal positive emotions. In other words, instead of evidence that both high‐arousal emotions were linked to preference for solitude, we found that preference for solitude was predicted by only the unpleasant kinds.

There are several things to consider when we interpret the above finding. First, we could not determine causal direction; that means, the association between high‐arousal negative emotions and preference for solitude could be bidirectional. Specifically, increase in high‐arousal negative emotions may trigger the preference for solitude or, alternatively, the preference for solitude may increase the experience of high‐arousal negative emotions. There were some experimental evidence suggesting that negative social experience that could trigger high‐arousal negative emotions, like being social excluded or ostracized, subsequently increased preference for solitude (Ren et al., 2016, 2021), but there has not been any evidence supporting the reverse direction.

We did originally anticipate that the effect of high‐arousal emotions on the likelihood of or preference for solitude would apply only for the negative kinds. But, along with the evidence showing that preference for solitude could be triggered by negative social experience (Ren et al., 2016, 2021), these findings suggest that it could be general negative emotions, in this case those high on arousal, that also trigger preference for solitude. Additionally, our results apply specifically to situations when the participants were spending time with others and experienced high‐arousal negative emotions. The link between high‐arousal negative emotions and preference for solitude is also more salient for younger people but not for older people. We interpreted these findings as meaning that, for younger adults, solitude might represent a space for how those negative emotions are dealt with. The heightened preference for solitude when experiencing negative emotions could reflect the desire to use solitude to process those emotions. This interpretation is built on the assumption that this preference for solitude is motivated by a self‐determined motivation (Thomas & Azmitia, 2019) to use time alone constructively for the purpose of mood regulation. This assumption is supported by the literature suggesting that some young people, particularly between the age of 18 and 25, find solitude useful for introspection (Long et al., 2003; Thomas, 2023). However, another interpretation is also possible: if negative emotions are symptoms of underlying social difficulties or psychological problems, this preference for solitude could be motivated by a non self‐determined motivation to be alone to avoid other people (Thomas & Azmitia, 2019), which could potentially lead into social isolation if prolonged. As such, it is important to distinguish the concept of preference for solitude from the motivation behind such desire (Nguyen et al., 2021); preference for solitude has been showed to correlate positively with both types of motivation (Thomas & Azmitia, 2019). To understand what drives the preference for solitude when one experiences negative emotion, more research is needed to hone into what younger people do when they are alone in a bad mood.

One reviewer at Stage 1 suggested that we also look at whether being alone would be associated with lower high‐arousal emotions. We found that solitude was associated with lower levels of high‐arousal positive emotions but there was no significant difference for high‐arousal negative emotions. Lower levels of high‐arousal negative emotions in solitude compared to social situations only showed up for younger but not older adults. This finding deviates from the “deactivation effect” discovered in previous studies that measured changes in high‐arousal emotions before and after participants, mainly university undergraduates, sat alone for 15 min in an experimental design (Nguyen et al., 2018). It is unclear whether this deviation between our findings with previous experimental findings might be due to differences in design, with previous ones looking at within‐person changes in high‐arousal negative emotions while our findings relying on correlational data.

Opposite to the findings for high‐arousal negative emotions, the difference in high‐arousal positive emotions between solitude versus social situations was driven by data from younger participants in our sample. The effect becomes smaller as age increases, which is consistent with the findings reported by Pauly et al. (2017). Note that Pauly et al.'s (2017) sample included participants that were older than our sample, and they found other age differences in low‐arousal emotions that we did not measure in our study.

Central to our research was the hypotheses that personality traits, specifically extraversion and neuroticism, would moderate the effects reported above. We did not find evidence that those personality traits moderated the link between high‐arousal emotions and solitude‐seeking or preference, nor was there a moderation effect of personality on the effect of being alone on emotional states. We found that people with higher neuroticism scores were more likely to prefer being alone whereas those with higher extraversion scores preferred social situations more. We did not find evidence that high‐neuroticism or low‐extraversion people actually reported more situations when they were alone. As such, our findings reflect associations between personality and high‐arousal emotions with subjective perceptions of preferring solitude, rather than self‐reports of being alone. As we previously discussed in the Introduction, there could be situational factors that prevented people in our sample to seek out solitude at the moment they might have preferred doing so. The limitation of only looking at changes in situation (from alone to social situation or vice versa) between adjacent time points (1 h apart) is that we might have failed to detect changes that might take longer to happen. For example, someone who might prefer to be alone at 11 am might not be able to find time to be alone until a few hours later because, for example, their work require that they interact with people. It is likely also that the time it takes between an emotional episode and the person's taking out time to be alone will vary across individuals and depend on the day of data collection. So, it might be more practical to have participants choose days when they are freer in movement (such as during the weekends or the holidays). While it could be argued that the weekends or the holidays may not be representative of behaviors on other days, it allows us to establish internal validity of the link between high‐arousal emotions and likelihood of being alone versus others.

### 5.1 Limitations

There are three issues that could be improved in future studies. First, age plays an important role in many of the effects we discovered in this data, but we had fewer participants in our study that were above the age of 30 (*N* = 87). While these participants provided a total of 1027 data points, future research would need to recruit more older participants to obtain more representative data for older age groups. Second, we did not ask the participants about the specifics of the situations where they reported high‐arousal negative or positive emotions. For example, previous research has showed that experiencing ostracism or social exclusion could lead to increased preference for solitude. As such, it was not clear whether our findings simply replicated that finding or whether it was the general experience of high‐arousal negative emotions that may trigger the desire for solitude. Data on what could have triggered those emotions could be useful for teasing that apart. Finally, the absence of differences on high‐arousal negative emotions between situations of being alone versus with others could be confounded by participants' motivation and attitude for solitude. Previous research suggested that motivation and attitude for solitude could moderate the effect of solitude on its deactivation effect (Study 4; Nguyen et al., 2018). Therefore, given that we did not find evidence supporting the roles of neuroticism and extraversion, future research could look at the moderation role of motivation for solitude (Nguyen et al., 2019; Thomas & Azmitia, 2019).

## 6 CONCLUSION

Overall, this research builds upon previous studies investigating the link between solitude and arousal regulation. Specifically, it examines whether individuals experiencing high‐arousal emotions are inclined toward seeking solitude or preferring solitude over social interactions. Although high‐arousal emotions did not predict actual reports of being alone, this could be because people often have little choice in navigating social contexts and obligations. However, we found that high‐arousal negative emotions are associated with a heightened preference for solitude, while high‐arousal positive emotions yield the opposite effect. We clarified these effects by showing that participants generally preferred solitude when they experienced high‐arousal negative emotions, but more so when they were with others at the time. So, it was not the case that people who experienced those negative emotions while alone would want to get out of their solitude to be with other; instead, they still preferred to remain in that state. On the other hand, individuals experiencing high‐arousal positive emotions generally displayed a greater preference for social interactions. Furthermore, we also looked at how these patterns changed depending on the participants' ages; the preference for solitude in response to high‐arousal negative emotions was more salient among younger compared to older individuals. These findings may suggest a distinctive appeal of solitude as a space for younger adults to process difficult emotions. This means that future research should hone into what factors will help younger adults to successfully regulate those emotions.

## AUTHORS CONTRIBUTIONS

Thuy‐vy T. Nguyen conceptualized, collected data, conducted the analyses. Delali Konu contributed to writing analysis code. Forbes checked code and model performance, as well as made recommendations to changes in model specifications. Thuy‐vy T. Nguyen drafted the manuscripts, Delali Konu and Forbes helped with review and revision of the final manuscript.

## FUNDING INFORMATION

The author(s) disclosed receipt of the following financial support for the research, authorship, and/or publication of this article: Preparation of this manuscript was supported by New Investigator Grant ES/W002256/1 from the Economic and Social Research Council.

## CONFLICT OF INTEREST STATEMENT

The author(s) declared no potential conflicts of interest with respect to the research, authorship, and/or publication of this article.

## ETHICS STATEMENT

We received ethics approval from Durham University's Psychology department ethics sub‐committee on December 19, 2022.

## Supporting information


**Data S1:** Supporting Information.
