# Supplementary material for: Investigating solitude as a tool for downregulation of daily arousal using ecological momentary assessments
Source: J Pers. 2024 May 17;93(1):31–50. doi: 10.1111/jopy.12939 (PMC11705511; doi:10.1111/jopy.12939)
Supplement: Supplementary file 1 — Data S1: Supporting Information. [file JOPY-93-31-s001.docx]

| *Table SM1.* Results of Linear Regression Testing the Interaction of Number of Surveys and High-Arousal Negative Emotions Predicting Proportion of Solitude Situation | | | | |
| --- | --- | --- | --- | --- |
|  | b | SE | z | *p* |
| (intercept) | 60.34 | 10.33 | 5.84 | <.001 |
| Number of surveys submitted | -1.50 | .83 | -1.81 | 0.071 |
| High-arousal negative emotions | .25 | 3.16 | .08 | 0.938 |
| Number of surveys x high-arousal negative emotions | .19 | .26 | .74 | 0.462 |

| *Table SM2.* Results of Mixed-Effects Logistic Regression Testing Whether the Situation that People were in (Solitude vs. Social Situation) Predict their Preference for Solitude vs. Social Situation | | | | |
| --- | --- | --- | --- | --- |
|  | b | SE | z | *p* |
| (intercept) | -.95 | .09 | -10.15 | <.001 |
| Situation | 2.37 | .11 | 22.52 | <.001 |
| *Notes. Current preference is used as outcome variable (solitude vs. social situation). Current situation is added as predictor. Random effects include random intercept and random slope of situation. The model uses ‘family = binomial("logit")’.* | | | | |

| *Table SM3.* Results of Linear Mixed-Effects Regression Testing the Interaction of Time (both Linear and Quadratic terms) and Current Situation (Solitude vs. Social Situation), Predicting Current High-Arousal Emotions (Negative or Positive Emotions) | | | | | | | | | | | | | |
| --- | --- | --- | --- | --- | --- | --- | --- | --- | --- | --- | --- | --- | --- |
|  | **Predicting high-arousal negative emotions** | | | |  | | **Predicting high-arousal positive emotions** | | | | |  |  |
|  | *b* | *SE* | *t* | *p* | |  | | *b* | *SE* | *t* | *p* |  |  |
| (intercept) | -.01 | .02 | -.31 | 0.756 | |  | | .10 | .01 | 5.07 | <.001 |  |  |
| **Within-person effects** |  |  |  |  | |  | |  |  |  |  |  |  |
| Time (linear) | -12.72 | 1.67 | -7.60 | <.001 | |  | | 7.04 | 1.83 | 3.86 | <.001 |  |  |
| Time (quadratic) | -.16 | 1.68 | -.10 | 0.923 | |  | | 5.39 | 1.65 | 3.26 | 0.001 |  |  |
| Situation at (t) | .02 | .03 | .76 | 0.445 | |  | | -.21 | .03 | -7.44 | <.001 |  |  |
| **Interaction** |  |  |  |  | |  | |  |  |  |  |  |  |
| Time (linear) x Situation at (t) | 2.36 | 2.01 | 1.17 | 0.241 | |  | | -6.88 | 2.02 | -3.40 | <.001 |  |  |
| **Time (quadratic) x Situation at (t)** | **2.67** | **1.99** | **1.34** | **0.180** | |  | | **-4.04** | **1.98** | **-2.04** | **0.041** |  |  |
| *Notes. Two separate models are presented here side by side, one predicting negative emotions in the left and one predicting positive emotion in the right. Each model includes the following fixed effects: linear and quadratic terms of time, current situation at time (t), and the interaction between personality trait and current situation. Random effects include random intercept and random slope of linear and quadratic terms of time. Linear and quadratic time components are orthogonal. Maximum likelihood estimations are used.* | | | | | | | | | | | | |  |

*Figure SM4.* Graph showing Changes in Current High-Arousal Emotions, depending on Current Situation (Solitude vs. Social)

*
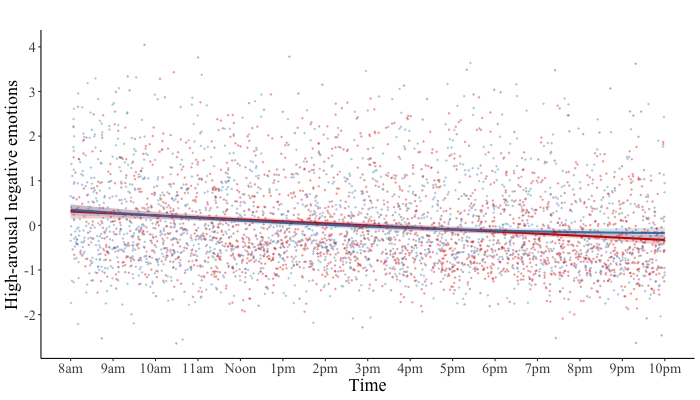
*

*
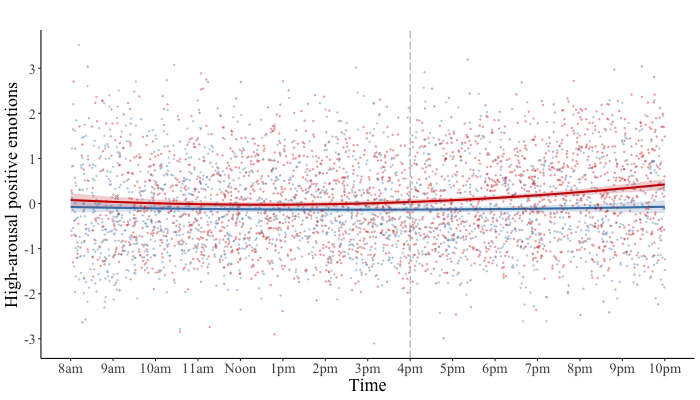
*

*Notes. Red line represents social situation; blue line represents solitude situation; dashed grey line represents value on the moderator (time) where there is a significant difference between solitude versus social situation on high-arousal positive emotions.*

*Figure SM5.* Proportions of Solitude Situations (vs. Social Situations) Reported at Each Hour of Assessments Throughout the Day.

*
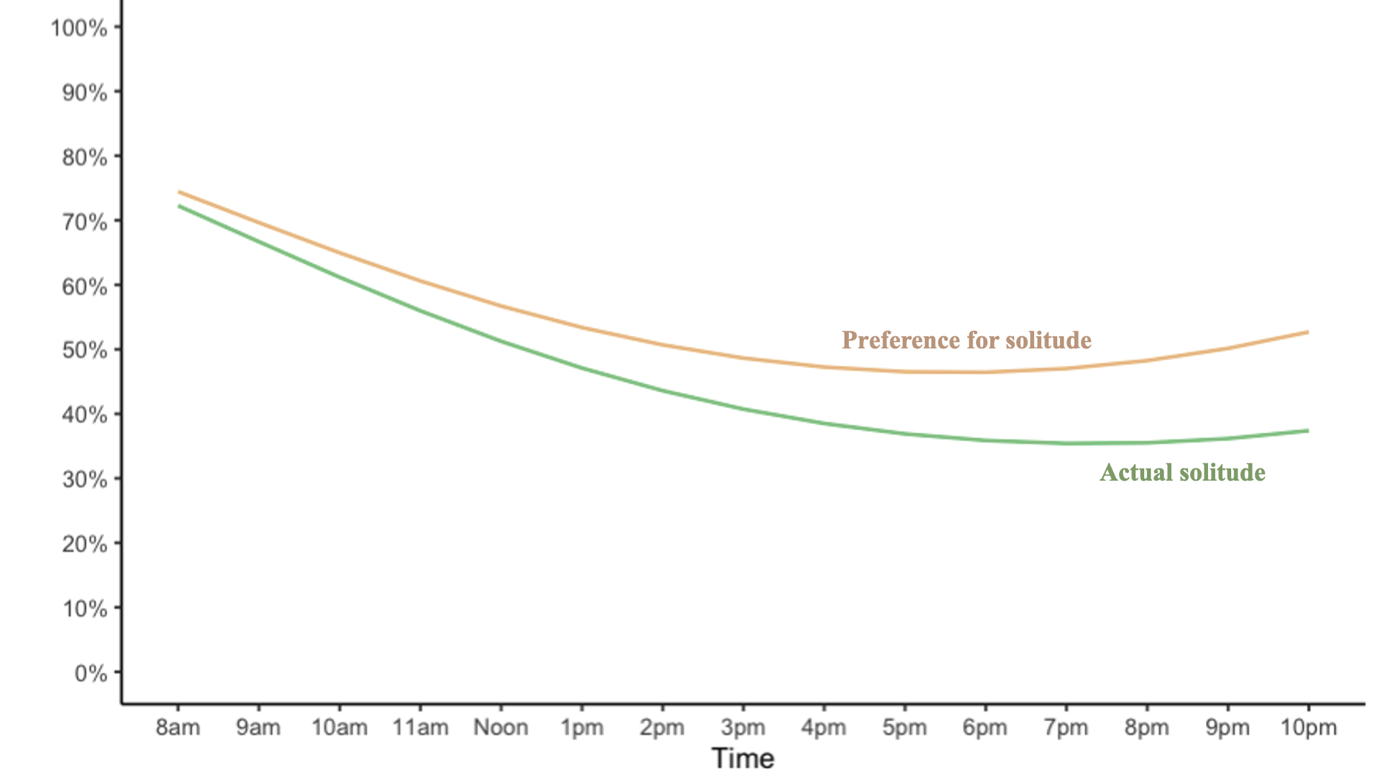
*

*Notes. Green line represents participants’ reports of whether they were alone or with other people; orange line represents participants’ reports of whether they preferred being alone or being with other people.*
